# Precise Droplet Dispensing in Digital Microfluidics with Dumbbell-Shaped Electrodes

**DOI:** 10.3390/mi13030484

**Published:** 2022-03-20

**Authors:** Wei Wang

**Affiliations:** 1 MOE Key Laboratory of Material Physics and Chemistry under Extraordinary Conditions, School of Physical Science and Technology, Northwestern Polytechnical University, Xi’an 710129, China; wangv@nwpu.edu.cn; 2 State Key Laboratory of ASIC and Systems, Fudan University, Shanghai 200433, China

**Keywords:** digital microfluidics, electrowetting, dumbbell-shaped electrodes, droplet dispensing, volumetric accuracy

## Abstract

Electro-wetting-on-dielectric (EWOD) enables the manipulation of droplets on a two-dimensional surface, which provides a versatile technique for digital microfluidics at a micro- or nano-scale. However, the deficiency of the dispensing precision has long limited its applications in micro total analysis systems (μ-TAS) where the accuracy of assays is largely determined by the volume control of the reagent dosing. This paper proposes optimum electrode designs and carries out characterization experiments to demonstrate the reproducibility of on-chip droplet generation with no extra external apparatus. The coefficient variation of the volumes of consecutively dispensed droplets from a non-refilling reservoir can be limited to below 0.3%, indicating the validity of the new electrode structure in practical applications.

## 1. Introduction

The lab-on-a-chip (LOC) concept, which refers to the miniaturization of traditional laboratory procedures, calls for the integration of standard reagent-based operations into a portable device. The accuracy of LOC platforms for micro total analysis systems largely depends on the volume control of the reagents dosing [1]. The development of microfluidic technologies utilizing micro/nano-channels promotes the handling of liquid with a pico- to milli-liter volume. However, the precise manipulation of the continuous flow relies on on/off-chip pumps and valves, which greatly hinders the integration and minimization of the total LOC systems [2,3,4,5,6]. Thus, a digital microfluidic technology that could control the motion of each individual droplet in the system has been proposed and developed instead.

**Figure 1:**
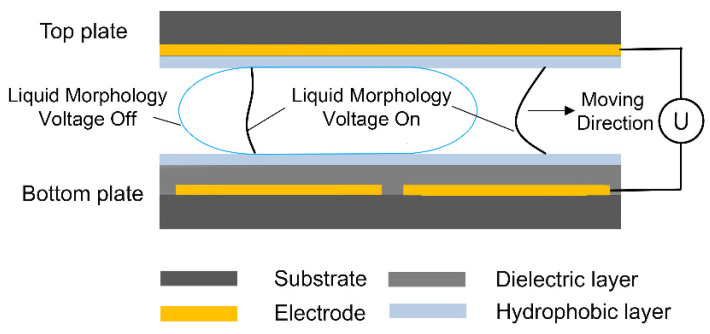
Schematic for electrowetting-on-dielectric (EWOD) principle. The light blue line demonstrates the droplet shape without applied voltages. The black line shows the droplet shape with the applied voltage. The applied voltage drives the liquid to the position of the actuated electrode.

**Figure 2:**
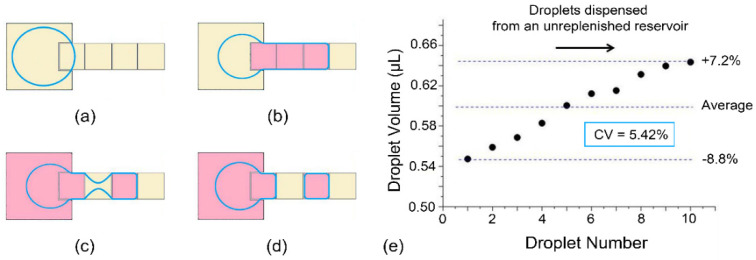
Droplet dispensing in traditional EWOD devices. (**a**–**d**) Schematic of the dispensing process. The blue contour demonstrates the shape of the liquid. The pink electrodes are applied to the actuation voltage, while the yellow ones are grounded. (**e**) Volume of droplets dispensed successively in traditional EWOD devices. Each dot is averaged over 5 measurements, and error bars are not shown for visual clarity.

Digital microfluidics based on electro-wetting-on-dielectric (EWOD) is a promising technique to manipulate droplets across a pre-designed routine in either single- or two-plate geometries. The precision of reagent dosing handling is one of its particularly useful properties, as reagents-contained droplets can be dispensed and manipulated reliably and precisely on-demand, catering to the need of micro total analysis systems and LOC applications [7,8]. In the two-plate format, dielectric and hydrophobic materials are coated on an array of patterned electrodes on both bottom and top plates, as shown in Figure 1. The active dispensing of droplets is realized by the proper control of an electrode actuation sequence in order to stretch, neck, and finally pinch a droplet off from the reservoir, as shown in Figure 2a–d. A higher voltage and larger force than movement action is required to act on the interface of the contact line to dispense a droplet from the reservoir due to the need to break the liquid stretched from the reservoir into two daughter parts. While the existence of fluidic instability in the actual world is essential to inducing the liquid pinch-off process, this process of unsteady states leaves the generating process extremely vulnerable to many uncertainties like the quality of manufacture and external disturbances. The dispensed volume is difficult to precisely control as it is affected not only by the manufacture quality (e.g., surface roughness, hydrophobic coating, dielectric layer properties, as well as their time dependency) and operation parameters (e.g., actuation sequence, driving voltage, or signal duration), but also by many random parameters [9]. Thus, the basic EWOD unit is not capable of generating droplets with the volumetric accuracy and consistency needed in many high-precision applications unless parameters are designed specifically for every application [10,11].

To improve the reproducibility of EWOD-based volume handling, off-chip pressure sources are sometimes adopted as an option to assist droplet dispensing [12]. A droplet could be formed on selected electrodes that remain active while the liquid is retracted, and EWOD electrodes are then actuated to move the droplet away. The volume variation of droplets dispensed in this work is reduced to about 5%. The system uncertainty could be further eliminated by incorporating the on-chip droplet volume metering mechanism [13] or feedback system [9], which further decreases the volume variation to about 1%. However, the extra pump or feedback setup compromises the simplicity of EWOD-based digital microfluidics systems, which is considered one of the greatest strengths when compared with other microfluidic techniques. Thus, an alternative function called passive dispensing was recently described by Wheeler et al. [14]. Hydrophilic sites were built on a hydrophobic surface to help the formation of droplets, but difficulties can be predicted for multi-step processes since the droplet gets stuck after dispensing. Therefore, it is difficult for the EWOD device to guarantee the generation of droplets with volumetric accuracy and consistency while maintaining the simplicity of EWOD systems at the same time.

Thus, this work proposes optimum electrode designs for EWOD systems to significantly improve the stability and reproducibility of the droplet dispensing process with no peripherals other than electrical connections. The work presented herein provides a detailed study of droplet dispensing, which provides a promising approach to the integration of micro total analysis systems with high liquid handling accuracy.

## 2. Materials and Methods

### 2.1. EWOD Chip on ITO Glass

As shown in Figure 1, indium tin oxide (ITO, 10 Ohms/sq)-coated soda-lime glass was used as the substrate for the EWOD structure following the fabrication procedure described elsewhere [15]. The ITO coating on bottom substrates was patterned with lithography and wet etching into designed two-dimensional electrode arrays. In this work, the actuation electrodes were designed as 2 × 2 mm with an 8 × 8 mm reservoir. A 2 μm SU-8 2002 photoresist and 60 nm Teflon-AF 1600 were then spin-coated on the surface as a dielectric layer and a hydrophobic coating, respectively. The top plate was fabricated by spin-coating 100 nm Teflon-AF 1600 on intact ITO glasses. The top and bottom plates were assembled parallelly using double-sided sticky tapes. Given that the droplet can only be successfully dispensed with a gap height smaller than a critical value [16,17], the gap width was adjusted by changing the number of tape layers. Moreover, considering the simplicity of fabrication and operation, the frequency of actuation supplies remains constant (1 kHz), and the medium filling the gap is air in this work.

### 2.2. Measurement of Droplet Volumes

To characterize the accuracy of the droplet volume, an image of the EWOD chip was recorded with a CCD camera immediately after dispensing. Pixels in the two-dimensional area of the dispensed droplet were counted with the aid of image processing software to estimate its top-view 2D area. The height of the gap between two plates was calculated from the layers of tape. Multiplying the gap height by the estimated 2D area gave an estimation of the droplet volume.

Reproducibility was measured by the coefficient of variation (CV) of the volumes of consecutively dispensed droplets, which could be calculated by the standard deviation (SD) divided by the mean value (MN). For droplets dispensed consecutively in the same characterization experiment, as the gap height remained constant, the CV could be derived directly from the variation of pixels in the 2D area for most conditions without multiplying the gap height. The use of CV makes the reproducibility of droplets with different mean volumes comparable.

## 3. Results

### 3.1. Droplet Dispensing with the Conventional Electrowetting Electrodes

The schematic of the breaking process on conventional EWOD devices is illustrated in Figure 2a–d, where the extracted liquid is pulled back to the reservoir, and the radius of the neck decreases until it is finally pinched off to generate a droplet. To investigate the volume variation of droplets, experiments were designed with the most widely used conventional electrode arrays, and the results are demonstrated in Figure 2e. Here, in characterization experiments with an 80 μm gap height, dispensing was attempted 10 times, as this was the maximum that could be dispensed steadily from a reservoir without re-filling. As the reservoir shrank until it was nearly empty, the droplet volume dispensed at 60 V_RMS_ increased by about 16% relative to the average volume, and the CV reached 5.42%. The monotonous increase of the droplet volume comes mainly from the decrease of the force drawing liquid back to the reservoir.

In practical conditions when the curvatures and voltages are time-dependent, dynamic statuses can be analyzed by two-dimensional hydrodynamic equations. The volume change at the creation site in the pinch-off process can be expressed as a function of the flow rate from the breaking electrode to the creation site. Consequently, the dispensed droplet volume can be calculated by integrating this volume change from the necking start until the droplet pinch-off occurs [9].

Ideally, to improve the reproducibility of the droplet volume, the parameters in this droplet dispensing process should remain constant for all steps. As the boundary of the liquid extruded from the reservoir meets that of the electrodes applied to the actuation voltage before necking starts, the initial extracted volume can be regarded as constant for droplets dispensed next to each other. Thus, the volume variation comes mainly from the necking process. Unfortunately, the electro-wetting force applied to the droplet is only a function of the applied voltage, the capacitance of the dielectric layer, and the projected length of the liquid boundary. As all other parameters remain constant in the dispensing process, the overall force induced by electro-wetting is in proportion to the length of the liquid boundary on the actuated electrode. When droplets were dispensed continuously without refilling the reservoir, the force pulling the liquid out of the reservoir remained almost the same as the volume of each dispensed droplet changed within a relatively small range, while the draw-back force rapidly decreased as the liquid in the reservoir shrank with the volume of droplets dispensed in this characterization experiment. Consequently, less liquid is drawn back to the reservoir, resulting in the rise of the dispensed droplet volume.

### 3.2. Square Sub-Electrode Design for Droplet Dispensing

While almost all parameters involved in the dispensing process can affect the droplet volume, the necking process is more important than other steps based on the analysis presented above. Since the volumetric inaccuracy of droplets dispensed consecutively from the non-filling reservoir is mainly due to the uncertainty of the whereabouts of liquids during the necking and splitting process, an intuitive solution is to reduce the size of the splitting electrode to reduce the volume of this portion of liquid [18,19].

**Figure 3:**
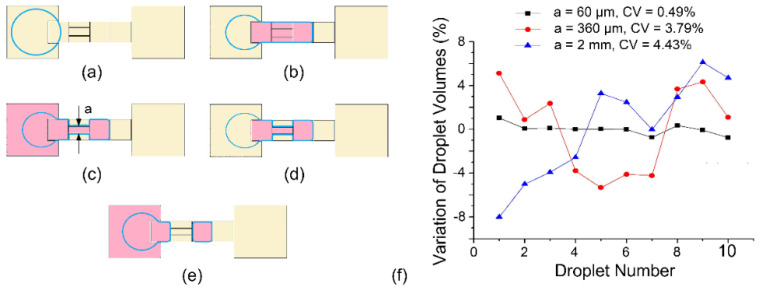
Droplet dispensing process with a new electrode design. (**a**–**e**) Schematic of the dispensing process. (**f**) Volume variation of droplets dispensed consecutively with the sub-electrodes of different widths. Each dot is averaged over 5 measurements, and error bars are not shown for visual clarity.

As illustrated in Figure 3a–d, the splitting electrode (on which the necking and splitting happen) is segmented into three sub-electrodes. Compared with the traditional microdroplet generation process shown in Figure 2, the necking process in Figure 3 is divided into two relatively controllable steps: the volume of liquid above the split electrode is first reduced by grounding the outer splitting sub-electrodes, and the necking process on the center sub-electrode starts after the remaining liquid gets stable. In this way, the volume of the liquid in an unstable necking state is significantly reduced from the entire splitting electrode to one sub-electrode. With a 160 μm gap height, the volume of consecutively dispensed droplets was tested experimentally. When the width of the center sub-electrode is 0.06 mm, 0.36 mm, or 2 mm, the experimental results are shown in Figure 3f. When the width of the sub-electrode decreases, the volumetric consistency of the dispensed droplets gradually increases. When the width of the sub-electrode is 0.06 mm, the coefficient of variation of the droplet volume decreases to about 0.49%, which is only about 9% of the CV of the traditional electrode structure (5.42%, as shown in Figure 2). However, factors such as the manufacturing difficulties, the surface tension of the liquid, and the contact angle saturation in electrowetting restrict the further decrease of the sub-electrode size. Narrow sub-electrodes will not only increase the difficulty of device fabrication but also reduce the stability of the device due to the need for a higher driving voltage.

### 3.3. Dumbbell-Shaped Sub-Electrode Design for Droplet Dispensing

**Figure 4:**
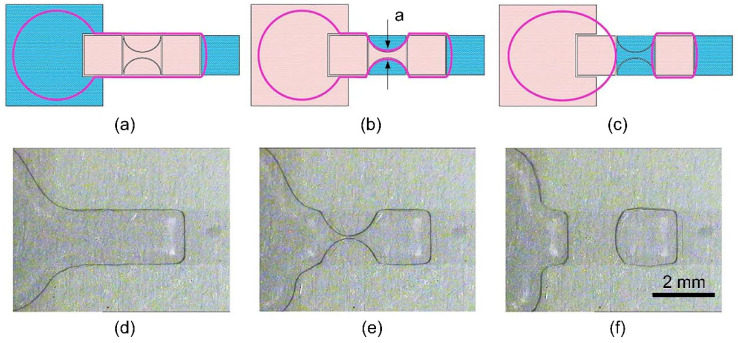
Schematics and photos of EWOD dispensing process with dumbbell-shaped electrode design (top view). (**a**–**c**) Schematic of the dispensing process. The pink electrodes are applied to the driving voltage with blue ones applied to the ground, and the purple circular contour indicates deionized water (DI water). a is the width of the finest point in the center dumbbell (denoted as neck width). (**d**–**f**) Photos of extrusion, necking, and breaking step, respectively.

Besides the whereabouts of liquids on splitting electrodes, the exact time and position of the pinch-off cannot be precisely controlled for traditional electrowetting electrode designs [20]. Herein, a new method is proposed to stabilize the droplet shape and restrict the position of the pinch-off. The illustration and experimental photos of the droplet dispensing process are shown in Figure 4. The new splitting electrode consists of two outer arcs and a center dumbbell. In this design, the cutting process to pinch off the liquid stretched from the reservoir is divided into two steps: (1) two outer arcs are at first connected to the ground to extract liquid from laterally to the media to form a stable neck on the center dumbbell-shaped sub-electrode, and (2) the center sub-electrode is turned hydrophobic after the liquid gets stable in order to pinch off at the predetermined location, namely the neck. Through this design, the EWOD chip improves the accuracy and consistency of droplet generation from the following aspects: (1) the introduction of sub-electrodes reduces the amount of liquid involved in the subsequent liquid shrinkage and necking process; (2) the profile of the dumbbell-shaped sub-electrode ensures that the liquid above it preferentially shrinks inward in the neck area under the effect of surface tension; (3) since the neck width of the sub-electrode can be extremely small, the reservoir only needs to suck back a very small amount of liquid to induce the pinch-off of liquid at the neck position.

**Figure 5:**
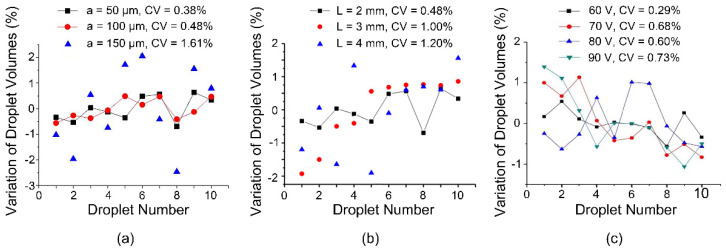
Volume variation of droplets as a function of (**a**) the neck width of the dumbbell-shaped sub-electrode, (**b**) the length of the dumbbell-shaped sub-electrode (the width remains 2 mm), and (**c**) the actuation voltage. Each dot is averaged over 5 measurements, and error bars are not shown for visual clarity.

Designs with different neck widths are fabricated with a 240 μm gap height for their effect on the droplet dispensing process. The results are demonstrated in Figure 5a. The droplet volume still oscillates for ten droplets dispensed from a non-replenishing reservoir, but its amplitude of variation and CV gradually decreases to ±0.5% and 0.38%, respectively, when the neck width is small enough (0.05 mm in this experiment). When the neck width is below 0.10 mm, the droplets dispensed consecutively maintain a high volumetric consistency (coefficient of variation less than 0.5%). When the neck width increases, the volumetric consistency drops rapidly until it reaches the same level as the traditional electrode design (as shown in Figure 2). As a large volume fluctuation is undesirable, the center dumbbell-shaped sub-electrode should be fabricated with a small neck in order to gain better reproducibility. With a properly chosen neck width, the pinch-off time and position can both be efficiently controlled. Moreover, the necking and pinch-off processes are greatly dependent on the capillary number, which is the ratio between the surface tension and the liquid viscosity. For conditions with small capillary numbers, the necking and pinch-off duration is elongated due to the eliminated Rayleigh–Plateau instability. Thus, the volumetric variation of the liquid in the reservoir has more influence on the volumetric variation of consecutively dispensed droplets.

**Figure 6:**
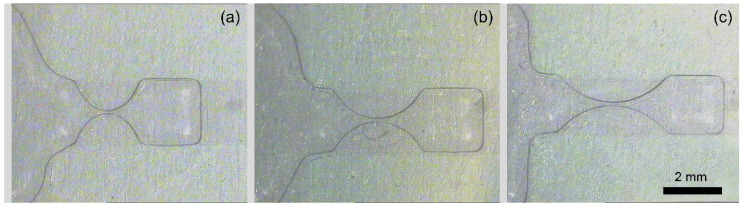
Photos of the liquid neck for the dumbbell-shaped sub-electrode with a length of (**a**) 2 mm, (**b**) 3 mm, and (**c**) 4 mm (the width remains 2 mm).

What is more, the length of the dumbbell-shaped sub-electrode is also changed to verify its effect on the droplet volume variation, and the results are shown in Figure 5b. Following the results obtained from Figure 5a, the neck width is set as 0.1 mm, and the length of the breaking electrode changes from 2 mm to 3 mm to 4 mm, while the width remains 2 mm, as demonstrated in Figure 6. When the length of the dumbbell-shaped sub-electrode gradually increases, the volumetric consistency of the generated water droplets gradually decreases. This result is mainly derived from two aspects: (1) the increase in the length of the dumbbell-shaped sub-electrode represents an increase in the volume of liquid above it during the splitting process; (2) the increase in the aspect ratio of the dumbbell-shaped sub-electrode reduces the curvature of the arcuate boundary and results in increased uncertainty in the pinch-off position. Even so, when the aspect ratio reaches 2, the volumetric consistency (CV = 1.20%) of the dispensed droplets is still much lower than the results when using conventional electrodes.

Moreover, the actuation voltage used in the above experiments is 60 V_rms_ sinusoidal alternating signals. Therefore, based on the classic Lippmann–Young equation, at the moment when the actuation voltage is applied or removed, the vibration amplitude of liquid will be different: the higher the actuation voltage, the more drastic the vibration of the liquid, the higher the instability in the liquid splitting process, and the worse the volumetric consistency of dispensed droplets. For this purpose, four sinusoidal voltage signals with a frequency of 1 kHz and an amplitude of 60 V_rms_, 70 V_rms_, 80 V_rms_, and 90 V_rms_ are experimentally tested. The length and width of the driving electrodes are both 2 mm. As shown in Figure 5c, the experimental results are roughly the same as in the analysis proposed above. Unexpectedly, the coefficient of variation does not increase monotonically with the increase of the magnitude of the actuation voltage, but increases first and then remains unchanged. It is believed that this is caused by the phenomenon of contact angle saturation. At the same time, since the coefficient of variation of droplet volumes has already been decreased to a very small value (when the driving voltage is 60 V_rms_, the coefficient of variation is 0.29%), even if the coefficient of variation at 90 V_rms_ has reached nearly three times that at 60 V_rms_, its absolute value is only 0.73%. Moreover, the excitation frequency of ac actuation voltages could also influence the volume variation due to its effect on the Rayleigh–Plateau instability, but thorough research of this effect is beyond the scope of this work.

## 4. Conclusions

Droplet dispensing is an essential step for digital microfluidics, as almost all further operations are carried out based on droplets and the volume of the droplet is determined in the dispensing process. In this paper, uncertainties in the droplet pinch-off process have been analyzed, and new dumbbell-shaped electrode designs are demonstrated without introducing an additional external apparatus. The reproducibility of the droplet volume falls in the range of ±0.5% for DI water when expanding the one-step breaking process into two steps: stable necking and final splitting. This all-electrical, simple, and efficient method demonstrates promising prospects for practical applications, bringing an improved uniformity of the droplet volume (about 10-fold smaller coefficient of variation compared to conventional ones with the same configuration). Besides, much more sophisticated theoretical work needs to be done in terms of the detailed modeling of the nonlinear droplet pinch-off process in order to improve the stability and robustness of the overall system.

## Data Availability

Not applicable.
